# Comparative analysis of 17 complete chloroplast genomes reveals intraspecific variation and relationships among *Pseudostellaria heterophylla* (Miq.) Pax populations

**DOI:** 10.3389/fpls.2023.1163325

**Published:** 2023-06-22

**Authors:** Wujun Zhang, Zhaolei Zhang, Baocai Liu, Jingying Chen, Yunqing Zhao, Yingzhen Huang

**Affiliations:** ^1^ Institute of Agricultural Bioresources, Fujian Academy of Agricultural Sciences, Fuzhou, China; ^2^ Hebei Key Laboratory of Study and Exploitation of Chinese Medicine, Chengde Medical University, Chengde, China

**Keywords:** *Pseudostellaria heterophylla*, chloroplast genome, comparative analysis, intraspecific variation, phylogenetic relationship

## Abstract

*Pseudostellaria heterophylla* (Miq.) Pax is a well-known medicinal and ecologically important plant. Effectively distinguishing its different genetic resources is essential for its breeding. Plant chloroplast genomes can provide much more information than traditional molecular markers and provide higher-resolution genetic analyses to distinguish closely related planting materials. Here, seventeen *P. heterophylla* samples from Anhui, Fujian, Guizhou, Hebei, Hunan, Jiangsu, and Shandong provinces were collected, and a genome skimming strategy was employed to obtain their chloroplast genomes. The *P. heterophylla* chloroplast genomes ranged from 149,356 bp to 149,592 bp in length, and a total of 111 unique genes were annotated, including 77 protein-coding genes, 30 tRNA genes, and four rRNA genes. Codon usage analysis showed that leucine had the highest frequency, while UUU (encoding phenylalanine) and UGC (encoding cysteine) were identified as the most and least frequently used codons, respectively. A total of 75–84 SSRs, 16–21 short tandem repeats, and 27–32 long repeat structures were identified in these chloroplast genomes. Then, four primer pairs were revealed for identifying SSR polymorphisms. Palindromes are the dominant type, accounting for an average of 47.86% of all long repeat sequences. Gene orders were highly collinear, and IR regions were highly conserved. Genome alignment indicated that there were four intergenic regions (*psaI*-*ycf4*, *ycf3*-*trnS*, *ndhC*-*trnV*, and *ndhI*-*ndhG*) and three coding genes (*ndhJ*, *ycf1*, and *rpl20*) that were highly variable among different *P. heterophylla* samples. Moreover, 10 SNP/MNP sites with high polymorphism were selected for further study. Phylogenetic analysis showed that populations of Chinese were clustered into a monophyletic group, in which the non-flowering variety formed a separate subclade with high statistical support. In this study, the comparative analysis of complete chloroplast genomes revealed intraspecific variations in *P. heterophylla* and further supported the idea that chloroplast genomes could elucidate relatedness among closely related cultivation materials.

## Introduction

*Pseudostellaria heterophylla* (Miq.) Pax (tai-zi-shen or hai-er-shen) is a well-known traditional medicinal plant of the Caryophyllaceae family. It is commonly used for the treatment of fatigue, spleen asthenia, anorexia, asthenia after severe illness, and cough due to lung dryness either in China (Commission, 2015) or in Korea (Liu et al., 2017). Recent pharmacologic research has indicated that *P. heterophylla* has anti-diabetes (Liu et al., 2017), immune enhancement (Yang et al., 2020), and anti-oxidant properties (Ng et al., 2004) due to its composition containing numerous active compounds such as cyclic peptides (pseudostellarin), polysaccharides, amino acids, saponins, and sapogenins (Wang et al., 2013). *P. heterophylla* is mainly distributed in the Fujian, Guizhou, Shandong, Anhui, and Jiangsu provinces of China (Kang et al., 2016), Japan, Korea, and the Russian Far East (Choi and Pak, 2000). *P. heterophylla* has been cultivated in China for over 100 years with abundant germplasm resources (Xiao et al., 2015), represented by significant variability in leaf length, leaf width, number of main stems, total biomass, and number of above-ground stem nodes. Currently, the breeding of *P. heterophylla* is progressing slowly since the introduction of varieties is not standardized and the genetic background of the cultivated populations cannot be traced. Moreover, there are few sexually reproduced varieties. However, long-term clonal reproduction is the main means of propagation in various regions, which leads to the erosion of the species genetic variability and restricts the development of utilization and applications (Wu et al., 2016). Therefore, finding a method that can distinguish different germplasm resources in *P. heterophylla* is urgent.

Previously, the chloroplast genome *rbcL* and *matK* regions, the Internal Transcribed Spacers (ITS) of the nuclear ribosomal DNA, sequence-related amplified polymorphism (SRAP), inter simple sequence repeat (ISSR), and expressed sequence tag-simple sequence repeat (ESR-SSR) have been used to characterize the genetic diversity of *P. heterophylla* germplasm (Yi et al., 2013; Xiao et al., 2014; Xu et al., 2023). Yi et al. (2013) found that the ITS sequences of different *P. heterophylla* varieties had several specific single nucleotide mutation sites and could be used to identify and distinguish samples from nine different producing areas. Xiao et al. (2014) used ISSR to analyze the diversity of 12* P. heterophylla* cultivars. A total of 73 polymorphic bands were identified, accounting for 89.02% of the total amplified bands, which revealed the clustering of these 12 cultivars into three clades.

With the development of high-throughput sequencing technologies and the decrease in sequencing costs, complete chloroplast genomes assembled from shotgun genomic DNA sequencing provide a more convenient and higher -resolution means to study the relationship among plant cultivated varieties (Straub et al., 2012). The chloroplast genome length is usually between 115 kb and 165 kb, and the length differences are mostly due to inverted repeat (IR) expansion/contraction (Zhu et al., 2016) or gene losses (Lei et al., 2016). As the second-largest plant genome, the chloroplast genome contains rich genetic information for species identification, phylogenetic analysis, and population genetic studies (Palmer, 1991). Dong et al. (2014) employed a chloroplast genomic strategy to design taxon-specific DNA mini-barcodes and applied them to species identification in the *ginsengs*. Liu et al. (2022) obtained chloroplast genome sequences of 24 plant samples in the genus *Atractylodes* and provided a new understanding of their phylogenetic relationship. Utilizing massively parallel sequencing technology for chloroplast genome sequencing in plants can facilitate a better understanding and discrimination of low-level systematic relationships among different taxa in plant classification (Parks et al., 2009). The first *P. heterophylla* chloroplast genome sequence distributed in Korea was reported and indicated that the *P. heterophylla* chloroplast genome has a double-stranded, circular, typically four-segment structure (Kim et al., 2019). However, there is still a lack of population genetic analyses in *P. heterophylla* using chloroplast genomes.

Here, we collected 17 P*. heterophylla* plant samples with remarkable phenotypic characteristics and obtained their chloroplast genome sequences using next-generation sequencing. This study aimed to (1) elucidate the conservation and diversity of *P. heterophylla* chloroplast genomes through comparative genomic approaches; (2) identify the most variable chloroplast genome regions to utilize them as markers for further germplasm conservation and genetic improvement; and (3) determine the relationships between genotypes using the chloroplast genome sequence data.

## Materials and methods

### Sample collection

In this study, 17 samples of *P. heterophylla* were collected from seven provinces and represented dominant cultivars in China (**Table 1**). Zheshen No. 1 has an erect growth habit, is unbranched and short, and its leaves are ovate. Its flowers are white, and its roots are spindle -shaped. It is moderately susceptible to leaf spot disease. Zheshen No. 2 has four to six upright branches (more than the Zheshen No. 1), ovate-lanceolate thick leaves, carrot-shaped root tubers, and moderate resistance to leaf spot disease. It does not flower. Zheshen No. 3 is a tetraploid *P. heterophylla* genotype induced by Zheshen No. 1. Zheshen No. 3 has oval, large, thick, dark green leaves, a low seed setting rate, and high-yielding roots. The Minxuan No. 6 and Minxuan No. 7 biotypes have long, oval, and thick leaves, flowering, large root tubers, and are more resistant to viral diseases. The Zherong Datiao was introduced from Guizhou and has characteristically large root tubers. Shitai No. 1 is a variety obtained using a mixed breeding approach. Its plants are upright, tall, and flowering, with round to long oval leaves and long spindle roots. The Guizhou cultivar plants are upright and tall, with oblong-ovate leaves and long spindle roots. The Jurong cultivar is a native cultivated variety with oval and thick leaves and high-yielding roots. The Hunan cultivar plants are upright, tall, and flowering, with long, ovate leaves and fusiform root tubers. The Xuancheng cultivar plants are upright and multi-branched and have tall plants with oblong-ovate leaves and large root tubers. The Shandong cultivar has been domesticated from a wild population. Its plants are tall with branches, and its leaves are oval-lanceolate and thin. The root tuber of the Shandong cultivar is long, spindle-shaped, and thin, and yields for this cultivar are high. The Hebei cultivar was introduced from Shandong and has morphological characteristics like the Shandong cultivar. These samples were identified by Prof. Jingying Chen from the Fujian Academy of Agricultural Sciences.

**Table 1:** Collection information of 17 P*. heterophylla* samples.

Sample ID	Cultivar name	Locality
TZ-1	Zheshen No. 1	Yingshan Town, Zherong County, Fujian Province
TZ-2	Zheshen No. 2	Fuxi town, Zherong County, Fujian Province
TZ-3	Zheshen No. 2	Fankeng Town, Fu ‘an City, Fujian Province
TZ-4	Zheshen No. 2	Shangbaishi Town, Fu ‘an City, Fujian Province
TZ-5	Zheshen No. 3	Yingshan Town, Zherong County, Fujian Province
TZ-6	Minxuan No. 6	Yingshan Town, Zherong County, Fujian Province
TZ-7	Minxuan No. 7	Chuping Town, Zherong County, Fujian Province
TZ-8	Zherong Datiao	Fuxi town, Zherong County, Fujian Province
TZ-9	Shitai No. 1	Niudachang town, Shibing County, Guizhou Province
TZ-10	Guizhou cultivar	Niudachang town, Shibing County, Guizhou Province
TZ-11	Jurong cultivar	Qianxu village, Jurong City, Jiangsu Province
TZ-12	Xuancheng cultivar	Zhongjianshan village, Guangde City, Anhui Province
TZ-13	Xuancheng cultivar	Jinshan Village, Guangde City, Anhui Province
TZ-15	Xuancheng cultivar	Sanhe Village, Xuanzhou District, Anhui Province
TZ-16	Hunan cultivar	Xiaoshajiang Town, Longhui County, Hunan Province
TZ-17	Hebei cultivar	Nanliu Town, Wuji County, Hebei Province
TZ-18	Shandong cultivar	Yushan Town, Linmu County, Shandong Province

### DNA extraction, library preparation, and high-throughput sequencing

The total genomic DNA from *P. heterophylla* leaf tissues was extracted using a modified CTAB method. DNA quantity and quality were determined using Qubit4.0 (Thermo Fisher Scientific Inc., USA). Subsequently, the genomic DNA was purified and fragmented to construct sequencing libraries (350 bp) using the TruSeq DNA PCR-Free High Throughput Library Prep Kit (Illumina, San Diego, CA). High-throughput sequencing (2 × 150 bp) was performed with the NovaSeq 6000 sequencer (Illumina, San Diego, CA).

### Assembly, annotation, and visualization of *P. heterophylla* chloroplast genomes

The PCR-free sequencing data were used to assemble the chloroplast genome sequences of *P. heterophylla* using the GetOrganelle pipeline (Jin et al., 2020). Gene annotation of the chloroplast genome sequences was performed using CpGAVAS2 (Shi et al., 2019) and then manually evaluated and corrected. Graphical maps of *P. heterophylla* chloroplast genome sequences were drawn using OrganellarGenomeDRAW (OGDRAW) (Greiner et al., 2019).

### Characterization and comparative analysis of *P. heterophylla* chloroplast genomes

The REPuter (Kurtz et al., 2001) software was used to recognize four types of sequence repeats, including forward (F), reverse (R), complementary (C), and palindromic (P). The minimum repeat size of oligonucleotide repeats was set at 30 bp, and the Hamming distance was set at 3. Tandem repeats were analyzed using the Tandem Repeats Finder (TRF) software (Benson, 1999) with default parameters. Simple sequence repeats (SSRs) were detected using the MIcroSAtellite identification tool (MISA) (Beier et al., 2017). The minimum repeat thresholds of mono-, di-, tri-, tetra-, penta-, and hexanucleotide SSRs were set as 10, 6, 5, 5, 5, and 5, respectively. Primers for SSRs were designed with Primer 3.0 software (Untergasser et al., 2012).

The mVISTA program with the Shuffle-Lagan model (Frazer et al., 2004) was employed to compare the chloroplast genome sequences of *P. heterophylla*. IRscope (Amiryousefi et al., 2018) was used to visualize the contraction and extension of IR boundaries between the four parts of the genome (LSC/IRb/SSC/IRa). Gene rearrangements were observed using the co-linear blocks obtained by the Mauve alignment algorithm (Darling et al., 2004).

ParaAT2.0 software (Zhang et al., 2012) was used to align protein sequences derived from specific protein-encoded DNA sequences extracted from 17 P*. heterophylla* chloroplast genomes. The nucleic acid alignment corresponding to the codon was translated back according to the protein alignment result. KaKs_Calculator 3.0 software (Zhang, 2022) was then used to calculate synonymous (Ks), nonsynonymous (Ka), and Ka/Ks ratios after homologous sequence alignment.

The concatenated protein-coding gene sequences of the 17 *Pseudostellaria* chloroplast genomes were used for phylogenetic analysis, with *Cerastium arvense*, *Gymnocarpos przewalskii*, and *Dianthus caryophyllus* as outgroup species. A maximum likelihood (ML) phylogenetic tree of 1,000 bootstrap replications was constructed using RAxML v8.2.12 (Stamatakis, 2014).

## Results

### Characterization of *P. heterophylla* chloroplast genomes

**Figure 1:**
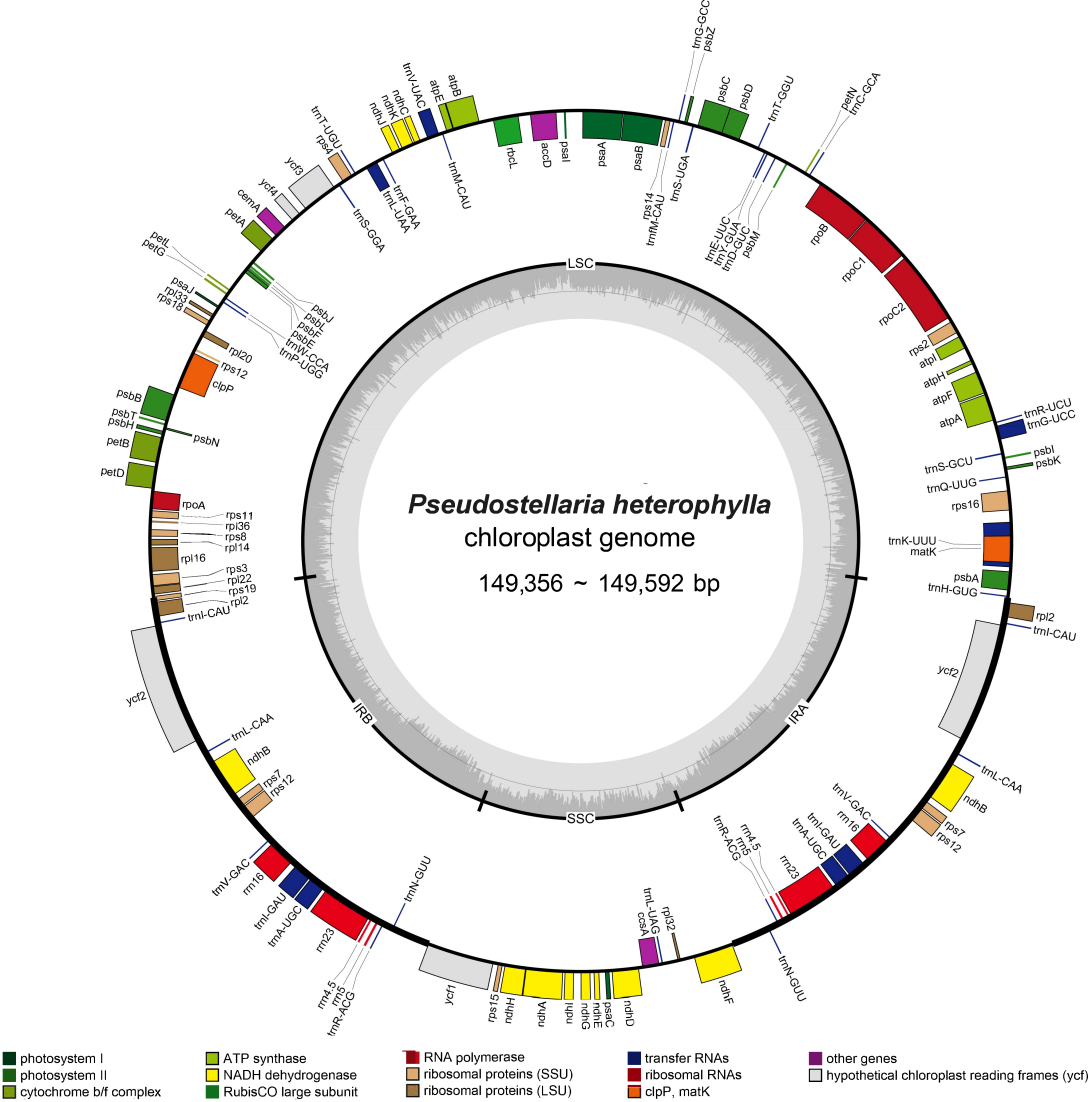
Circular chloroplast genome map of *P. heterophylla*. Genes drawn outside the circle are transcribed clockwise, and those inside are counterclockwise. Genes belonging to different functional groups are color-coded. The dark gray area in the inner circle denotes GC content. LSC, large single copy; SSC, small single copy; and IR, inverted repeat.

The *P. heterophylla* chloroplast genome sequence length ranged from 149,356 bp to 149,592 bp, with a variation of 236 bp among the different samples. Each chloroplast genome had the typical quadripartite structure, with a large single copy (LSC) region (80,994–81,144 bp), a small single copy (SSC) region (16,860 to 17,154 bp), and a pair of IR regions (IRa and IRb) (25,650 to 25,732 bp). The chloroplast genome GC content in all samples ranged from 36.50% to 36.52%, and the GC content in the IR region (approximately 42%) was significantly higher compared to the LSC region and SSC region (approximately 34% and 29%). A total of 111 unique genes were annotated in the *P. heterophylla* chloroplast genomes sequenced, including 77 protein-coding genes, 30 tRNA genes, and four rRNA genes (rrn23S, rrn16S, rrn5S, and rrn4.5S). Among these genes, 46 were related to photosynthesis, and 58 were involved in chloroplast transcription and translation activities. Fifteen genes were in the IR region with two copies, including four protein-coding genes, seven tRNA genes, and four rRNA genes. Seventeen genes contained introns, of which 14 genes (eight protein-coding genes and six tRNA genes) contained one intron, and three genes (*rps12*, *ycf3*, and *clpP*) contained two introns. Small exons were also identified in the *petB*, *petD*, and *rpl16* genes, with lengths of 6 bp, 8 bp, and 9 bp, respectively. In addition, *rps12* was identified as a trans-splicing gene. Further detailed chloroplast genome information is presented in **Tables 2**, **S1** and **Figure 1**.

**Table 2:** Genes in the chloroplast genome of *P. heterophylla*.

Category	Group	Genes
Miscellaneous group	Acetyl-CoA carboxylase	*accD*
	Cytochrome c biogenesis	*ccsA*
	Maturase	*matK*
Photosynthetic genes	Subunits of ATP synthase	*atpA*, *atpB*, *atpE*, atpF*, *atpH*, *atpI*
	Chloroplast envelope membrane protein	*cemA*
	ATP-dependentprotease subumitP	clpP**
	Subunits of NADH dehydrogenase	ndhA*, ndhB*, *ndhC*, *ndhD*, *ndhE*, *ndhF*, *ndhG*, *ndhH*, *ndhI*, *ndhJ*, *ndhK*
	Subumits of cytochrome	*petA*, petB*, petD*, *petG*, *petL*, *petN*
	Subunits of photosystem I	*psaA*, *psaB*, *psaC*, *psaI*, *psaJ*
	Subunits of photosystem II	*psbA*, *psbB*, *psbC*, *psbD*, *psbE*, *psbF*, *psbH*, *psbI*, *psbJ*, *psbK*, *psbL*, *psbM*, *psbN*, *psbT*, *psbZ*
	The large subunit of Rubisco	*rbcL*
Transcription and translation-elated genes	Large subunit of ribosome	*rpl14*, rpl16*, rpl2, rpl20, rpl22, rpl32, rpl33, rpl36
	Small subunit of the ribosome	rps11, rps12**, rps14, rps15, rps16*, rps18, rps19, rps2, rps3, rps4, rps7, rps8
Protein synthesis and DNA replication	RNA polymerase	rpoA, rpoB, rpoC1*, rpoC2
RNA genes	Ribosomal RNA genes	*rrn16*, *rrn23*, *rrn4.5*, *rrn5*
	Transfer RNA genes	trnA-UGC*, *trnC-GCA*, *trnD-GUC*, *trnE-UUC*, *trnF-GAA*, *trnG-GCC*, trnG-UCC*, *trnH-GUG*, *trnI-CAU*, trnI-GAU*, trnK-UUU*, *trnL-CAA*, trnL-UAA*, *trnL-UAG*, *trnM-CAU*, *trnN-GUU*, *trnP-UGG*, *trnQ-UUG*, *trnR-ACG*, *trnR-UCU*, *trnS-GCU*, *trnS-GGA*, *trnS-UGA*, *trnT-GGU*, *trnT-UGU*, *trnV-GAC*, trnV-UAC*, *trnW-CCA*, *trnY-GUA*, *trnfM-CAU*
unknown function	Hypothetical chloroplast reading frames(*ycf*)	*ycf1*, *ycf2*, ycf3**, *ycf4*

^*^Contains one intron; ^**^Contains two introns.

### Codon usage in *P. heterophylla* chloroplast genomes

The amino acid frequencies, the number of codons, and the relative synonymous codon usage (RSCU) in *P. heterophylla* chloroplast genomes are shown in **Table S2**. The average RSCU value was 63.97, and the number of codons ranged from 22,012 (TZ-3) to 22,017 (TZ-5). Among the codons, leucine was the amino acid with the most abundant codons. UUU (encoding phenylalanine) and UGC (encoding cysteine) were the most and least used codons, respectively. Almost all amino acids had more than one synonymous codon, except for methionine and tryptophan. Four start codon types were identified in the 77 protein-coding genes. Among them, 73 genes possessed ATG as their start codon, while two genes (*ndhD* and *psbL*) had ACG, one gene (*rps19*) had GTG, and one gene (*ycf1*) had TTG as their start codon. All the samples had the same three stop codon types (TAA, TAG, and TGA). The most used stop codon was TAA (60.98%), followed by TGA (21.95%) and TAG (17.07%).

### SSRs, repeat structures, and IRs of *P. heterophylla* chloroplast genomes

**Figure 2:**
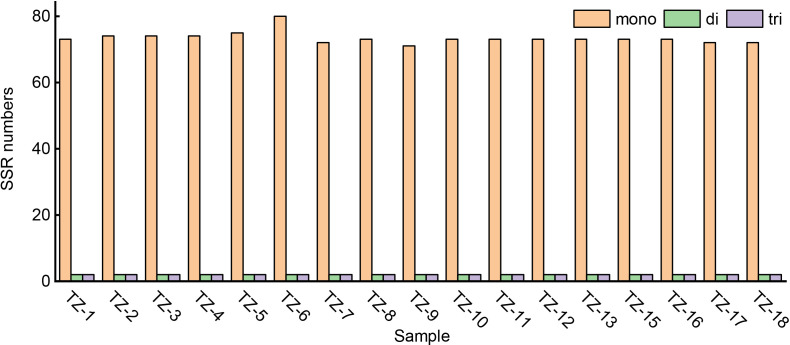
Simple sequence repeats (SSRs) in the chloroplast genome of *P. heterophylla*.

For the SSR analysis, 75–84 SSR loci were detected in the *P. heterophylla* chloroplast genomes (**Figure 2**), among which polyadenine (poly-A) (54.78%, 41–47) and polythymine (poly-T) (38.75%, 29–32) represented the most abundant simple sequence repeats. SSRs and their 500 bp upstream and downstream sequences were extracted, and 69 primer pairs were designed using Primer 3.0 software. After electronic amplification evaluation allowing for two mismatches, four pairs of SSR primers targeting highly polymorphic SSR regions were obtained (**Table S3**). Sixteen to 21 short tandem repeats were found in the *P. heterophylla* chloroplast genomes (**Table S4**), ranging in length from 11 to 32 bp, with most located in the intergenic space (IGS) regions. Twenty-seven to 32 long repeat structures were identified in the *P. heterophylla* chloroplast genomes, including forward, palindromic, reverse, and complement repeats (**Table S5**). Palindromic was the most common repeat sequence type, accounting for an average of 47.86% of all repeat sequences, followed by forward (40.12%), reverse (11.21%), and complement (0.81%).

The *P. heterophylla* chloroplast genome exhibits four boundaries between the IRs and LSC/SSC regions: LSC-IRb, IRb-SSC, SSC-IRa, and IRa-LSC (**Figure S1**). The LSC-IRb, IRb-SSC, SSC-IRa, and IRa-LSC boundaries in all samples were located at *rps19*, *ycf1*, *ndhF*, and *rpl2-trnH*, respectively. The *P. heterophylla* chloroplast genomes from the Chinese populations were highly conserved. The nucleotide lengths of *rps19* and *ycf1* located in the IRb region were 195 bp and 105 bp, of *ndhF* located in the IRa region was 56 bp, and of *trnH* from the IRa-LSC boundary was 29 bp.

### Candidate markers and Ka/Ks substitution of *P. heterophylla* chloroplast genomes

**Figure 3:**
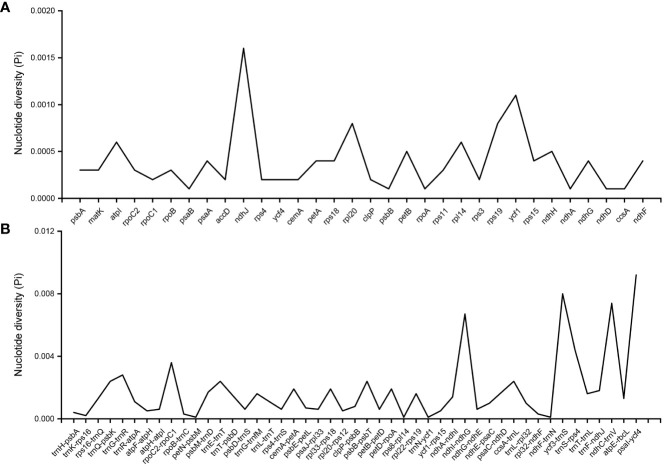
The nucleotide diversity of genes and intergenic regions in the *P. heterophylla* chloroplast genomes. **(A)** Coding region; **(B)** Noncoding region.

According to the comparative analysis of the whole chloroplast genome of *P. heterophylla* using the LAGAN program, several regions were variable and were able to distinguish different populations (**Figure S2**). In terms of genes, the most variable coding genes were *ndhJ*, *ycf1*, and *rpl20*, and the most variable intergenic regions were *psaI*-*ycf4*, *ycf3*-*trnS*, *ndhC*-*trnV*, and *ndhI*-*ndhG* (**Figure 3**). Among these genes and intergenic regions, *ycf1* and *ndhI-ndhG* contained a higher number of SNP and MNP polymorphic loci. Particularly, 10 highly polymorphic SNP/MNP loci were identified, which could be used as candidate SNP/MNP markers to distinguish different populations (**Table 3**). Then, the Mauve algorithm was used to identify the local collinear blocks (LCBs) of the *P. heterophylla* chloroplast genomes, with NC_044183 selected as the reference genome (**Figure S3**). Among all the chloroplast genomes of the samples, the collinear blocks, including the LSC, SSC, and IR regions, showed relatively high levels of conservation and no gene rearrangements. Thirty-two protein-coding genes with polymorphic sites were used to analyze the synonymous (Ks) and non-synonymous (Ka) substitution rates (**Table S6**). The average Ka value of the 15 genes was higher than 0.001 (**Figure S4**), with *rps15*, *rpoC2*, and *rpl20* exhibiting the highest Ka values. Meanwhile, the average Ks value of 17 genes, such as *rps19*, *rps18*, and *rpl14*, was higher than 0.001. The Ka/Ks ratio of all these 32 protein-coding genes ranged from 0.001 to 49.884, with an average value of 19.244. The Ka/Ks ratio of 15 genes was higher than 1, and the gene with the highest Ka/Ks ratio was *rps15* (49.88).

**Table 3:** Candidate polymorphic DNA markers from the chloroplast genome of *P. heterophylla*.

No.	Position*	Polymorphic Type	Variant	Location
1	743–876	SNP	A/T, G/T, T/G	*matK*
2	1,184–2,050	SNP	G/A, T/A, A/T, G/A, G/A	*ndhF*
3	420–900	SNP	A/T, G/C, G/T	*ndhH*
4	2,778–4,100	SNP	A/T, A/G, A/G, A/C	*rpoC2*
5	213–3,605	SNP/MNP	A/G, G/T, A/T, A/C, A/T, A/C, AA/CT, A/T, T/A, T/A, T/A, T/A, T/A, A/T, C/A, A/G, A/T, C/A, G/A, C/T, A/T, A/C	*ycf1*
6	67–247	SNP/MNP	T/A, CAAAATTT/ATTGTAGG, A/T, AA/TT, A/T, T/G	*ndhI_ndhG*
7	21–627	SNP	G/A, A/T, A/T, A/T, A/C, A/T, G/G	*rps16_trnQ-UUG*
8	13–299	SNP	C/T, A/T, A/T	*trnE-UUC_trnT-GGU*
9	7–340	SNP	G/T, C/A, T/A, T/G	*trnL-UAG_rpl32*
10	111–162	SNP	G/A, G/T, C/A	*trnT-GGU_psbC*

*Position is based on the gene and gene spacer alignment data. SNP, single nucleotide polymorphism; MNP, multiple nucleotide polymorphism.

### Phylogenetic analysis of *P. heterophylla* chloroplast genomes

**Figure 4:**
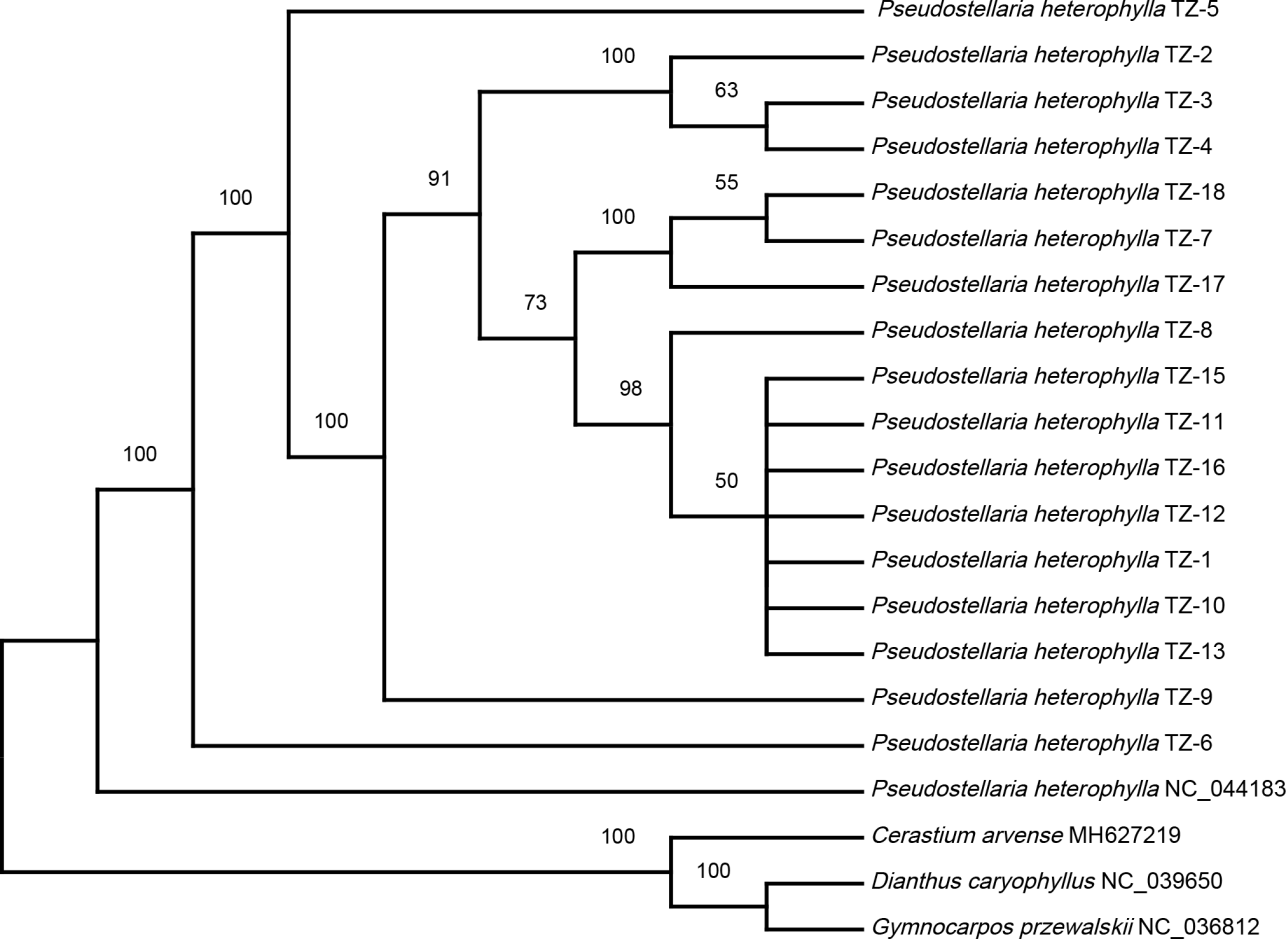
Maximum likelihood (ML) phylogenetic tree of *P. heterophylla* inferred from chloroplast genome sequences. Bootstrap values are shown at each node.

To explore the relationships among *P. heterophylla* cultivars, a maximum likelihood (ML) phylogenetic tree was constructed, and *C. arvense*, *G. przewalskii*, and *D. caryophyllus* were selected as out group species (**Figure 4**). The samples belonging to the Korean *P. heterophylla* population formed a separate cluster from the samples from the Chinese population. In terms of the Chinese *P. heterophylla* population samples, TZ-1, TZ-8, TZ-10–TZ-13, TZ-15, and TZ-16 were clustered into a big branch, which may be due to the mutual introduction of *P. heterophylla* from Fujian, Jiangsu, Anhui, Hunan, Guizhou, and other locations, resulting in a high similarity of the germplasm resources. TZ-17, TZ-18, and TZ-7 were clustered into a smaller branch that is related to Shandong *P. heterophylla* sources. Three samples of Zheshen No. 2 (TZ-2, TZ-3, and TZ-4) from different places were clustered into a separate branch. TZ-5, TZ-6, and TZ-9 were in separate branches that were located towards the edges of the phylogenetic tree. TZ-6 was selected for its virus resistance. The above results indicated that chloroplast genome sequence analyses could provide useful information for assessing the genetic background of a species. They could be used to assist breeding and provide a molecular –biological basis for cultivar identification.

## Discussion

Distinguishing germplasm resources is essential for plant breeding. Traditional breeding efforts in *P. heterophylla* have usually used plant morphological characteristics, such as leaf size, shape, and thickness; rhizome length, diameter, and texture; plant height; and the number of flowers, to distinguish varieties. However, phenotypes are easily affected by cultivation methods and environmental factors and require long-term observation (Chen et al., 2010). In addition, the irregular introduction of *P. heterophylla* has also impacted the distribution of *P. heterophylla* genetic resources, which affected the uniform collection, classification, and identification of germplasm resources (Xu et al., 2023). *P. heterophylla* cultivation has a history of more than 180 years, and at its earliest stage of cultivation, *P. heterophylla* germplasm resources were mainly derived from wild populations. Since the 1960s, Fujian has successively introduced resources from Jiangsu, Anhui, Zhejiang, Shandong, and other places that formed novel germplasm resources, such as Zheshen No. 1 and Zheshen No. 2. Guizhou Province has no wild *P. heterophylla* populations, and *P. heterophylla* was introduced from Fujian for cultivation in the 1990s. Wild resources of *P. heterophylla* are highly abundant in Jiangsu Province, where there are rarely germplasm introductions from other locations. Due to the use of seeds to raise seedlings, *P. heterophylla* in Jiansu Province is less affected by viral diseases and achieves a higher yield. Interestingly, phylogenetic analysis in this study has provided clues to trace the breeding history of these resources and further verified that the chloroplast genome can provide useful information for analyzing the genetic background of this species. The Korean population was located on the outermost part of the phylogenetic tree as an outgroup. Previous studies reported that *P. heterophylla* is distributed throughout the mountains of Korea, and the morphological characteristics of the Korean population are different from those distributed in China (Choi and Pak, 2000). Therefore, it is possible to obtain new genetic resources or special breeding materials by hybridizing the Korean population with Chinese populations based on the theory of distant hybridization.

Molecular markers derived from the chloroplast genome, such as *rbcL*, *matK*, and *psbA-trnH*, are effective for species identification and phylogenetic resolution (Shi et al., 2011; Liu et al., 2014), and several DNA barcode libraries have been established (Liu et al., 2017). However, species or biotype identification with molecular markers still faces many challenges, especially for closely related species and different populations within species. Previous studies demonstrated that the identification efficiency of DNA barcode markers in specific regions for closely related species was only about 80% (Chen et al., 2014). Several highly informative DNA barcode markers for specific taxa have been developed using comparative analyses of c hloroplast genomes (Zhou et al., 2022). After a comparative analysis of the *Rheum palmatum*, *R. tanguticum*, and *R. officinale* chloroplast genomes, five hypervariable regions (*rps16*-*trnQ*, *psaA*-*ycf3*, *psbE*-*petL*, *ndhF*-*rpl32*, and *trnT*-*trnL*) were identified and used as specific DNA barcodes for the identification of 42 samples among *R. tanguticum, R. officinale*, and *R*. *palmatum* (Li et al., 2022). The *trnl*-*GAU* intron region was detected to be highly variable and will be useful for future evolutionary studies, although the data from four widely distributed varieties were highly conserved (Wang et al., 2018). The chloroplast genome comparison of *Gentiana* species revealed that the six most InDel-variable loci could be selected as regions for DNA barcode genotyping, confirming that chloroplast genomes could improve the discriminatory capacity for species identification (Zhou et al., 2018). Seven regions (*rpl32-ccsA*, *rpl20-clpP*, *trnC-rpoB*, *ycf1b*, *accD-ycf4*, *ycf1a*, and *psbK-accD*) were identified from the *Pterocarpus* chloroplast genome by quantifying nucleotide diversity and had remarkably higher variability compared to the plant universal barcodes (*rbcL*, *matK*, and *trnH*-*psbA*) (Jiao et al., 2019). The comparison of the rose chloroplast genome revealed that 15 cpSSRs and 150 flanking single nucleotide variations (SNVs) exhibited higher divergence and stronger power for the genotyping of rose varieties (Li et al., 2020). Moreover, the chloroplast genome can also be used as a super-barcode for phylogenetic and closely related taxon identification studies (Chen et al., 2018).

## Conclusion

Using high-throughput sequencing approaches, we obtained the complete chloroplast genome sequences of seventeen *P. heterophylla* varieties. The gene contents and gene orders of the chloroplast genomes were highly conserved. Among these cultivars, 75–84 SSRs, 16–21 short tandem repeats, and 27–32 long repeat structures were detected. Four primer pairs were designed to target highly polymorphic SSR loci. Gene orders were collinear, and IR regions were conserved. Four intergenic regions and three coding genes were found to be highly variable, and ten SNP/MNP sites with polymorphisms were identified and selected for further study. Phylogenetic analysis showed that Chinese populations were clustered into a monophyletic group, in which the non-flowering varieties formed a separate subclade. This study verified that chloroplast genomes could elucidate the relationship among closely related cultivated materials and provide useful information for developing new, highly polymorphic, and informative molecular makers.

## Data availability statement

The original contributions presented in the study are publicly available. This data can be found here: NCBI, PRJNA932041. The GenBank numbers provided are: OQ405025.1~OQ405039.1, OK643505.1, and OK643506.1.

## Author contributions

WZ and JC conceived and designed the experiments. WZ and BL performed the experiments. WZ, ZZ, YZ, and YH analyzed and interpreted the data. WZ and ZZ wrote the manuscript. JC revised and approved the manuscript. All authors listed have made a substantial, direct, and intellectual contribution to the work and approved it for publication.

## Supplementary material

Supplementary Table 1 Genome features of *P. heterophylla* chloroplast genomes.

Supplementary Table 2 Codon usage of *P. heterophylla* chloroplast genomes.

Supplementary Table 3 Primer pairs for SSRs.

Supplementary Table 4 Tandem repeats in *P. heterophylla* chloroplast genomes.

Supplementary Table 5 Repeat structures in *P. heterophylla* chloroplast genomes.

Supplementary Table 6 Ka/Ks analysis of genes with variable sites.

Supplementary Figure 1 The comparison of the *P. heterophylla* chloroplast genome junction boundaries. JLB, junction of LSC and IRb; JLA, junction of LSC and IRb.

Supplementary Figure 2 The comparative analysis with LAGAN program of the whole-chloroplast genome of *P. heterophylla*. The x-axis represents the coordinate in the chloroplast genome.

Supplementary Figure 3 A comparison of the whole plastid genomes of *P. heterophylla* using the Mauve algorithm. The red LCBs indicate syntenic regions, while the histograms within each block represent the degree of sequence similarity. rRNA, protein-coding, and tRNA gene annotations are denoted by red, white, and green boxes, respectively.

Supplementary Figure 4 Ka/Ks analysis of *P. heterophylla* chloroplast gnomes. **(A)** Ka, rate of nonsynonymous substitution; **(B)** Ks, rate of synonymous substitution; **(C)** Ka/Ks, rate of non-synonymous vs. synonymous substitutions.
