# Supplementary material for: Comparative analysis of 17 complete chloroplast genomes reveals intraspecific variation and relationships among Pseudostellaria heterophylla (Miq.) Pax populations
Source: Front Plant Sci. 2023 Jun 22;14:1163325. doi: 10.3389/fpls.2023.1163325 (PMC10325831; doi:10.3389/fpls.2023.1163325)
Supplement: Supplementary Table 3 — Primer pairs for SSRs. [file Table_3.docx]

Table S3. Primer pairs for SSRs

| Primer ID | Primer | SSR | Tm | Target |
| --- | --- | --- | --- | --- |
| SSR_53 | Forward primer, ACGCCTCTGCATCTAGCATT  Reverse primer, TGCGCATTGAATTTCTGCTA | (T)12 | 60.4 | 136 |
| SSR_54 | Forward primer, TCCACCCAGTTTATCCATTTTC  Reverse primer, TTGGCAAATATTCCTTTTTCG | (A)10 | 60.0 | 245 |
| SSR_67 | Forward primer, TCGTTTTCTCATTGCATAATCG  Reverse primer, TCCACCCAGTTTATCCATTTTC | (T)10 | 60.1 | 227 |
| SSR_69 | Forward primer, ATGCTGGCGCAAAAGATACT  Reverse primer, AAATCGTGATTGGGATAGCG | (A)10 | 59.9 | 108 |
