# Supplementary figures and images for: Comparative analysis of 17 complete chloroplast genomes reveals intraspecific variation and relationships among Pseudostellaria heterophylla (Miq.) Pax populations
Source: Front Plant Sci. 2023 Jun 22;14:1163325. doi: 10.3389/fpls.2023.1163325 (PMC10325831; doi:10.3389/fpls.2023.1163325)

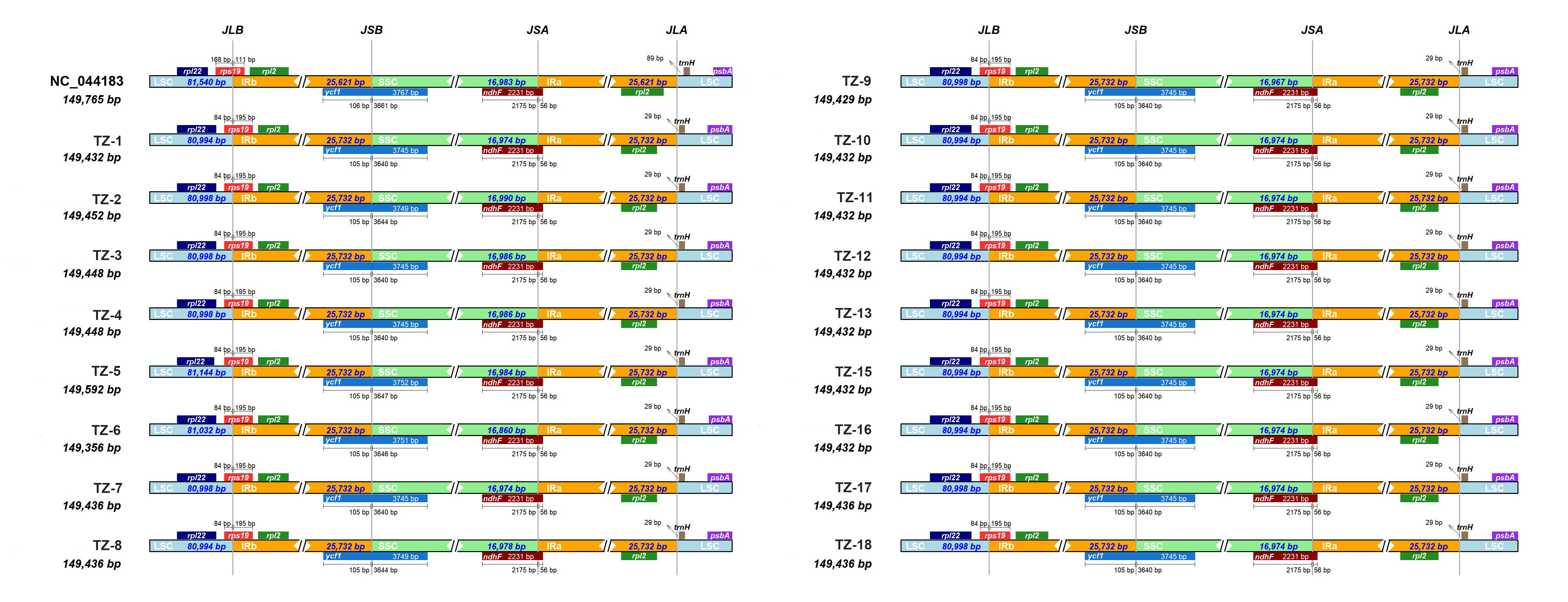

Supplement: Supplementary Figure 1 — The comparison of the P. heterophylla chloroplast genome junction boundaries. JLB, junction of LSC and IRb; JLA, junction of LSC and IRb. [file Image_1.tif]

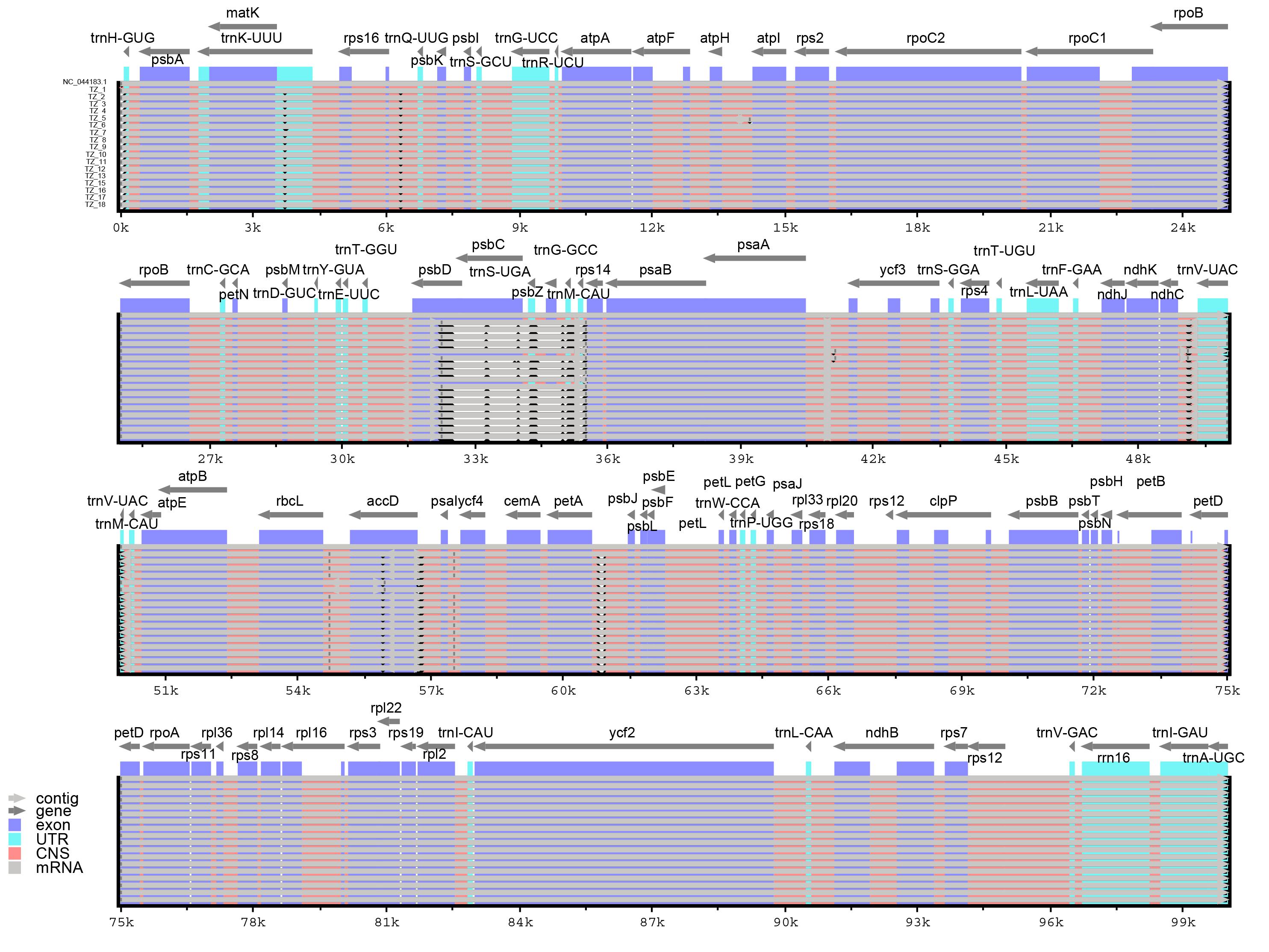

Supplement: Supplementary Figure 2 — The comparative analysis with LAGAN program of the whole-chloroplast genome of P. heterophylla. The x-axis represents the coordinate in the chloroplast genome. [file Image_2.tif]

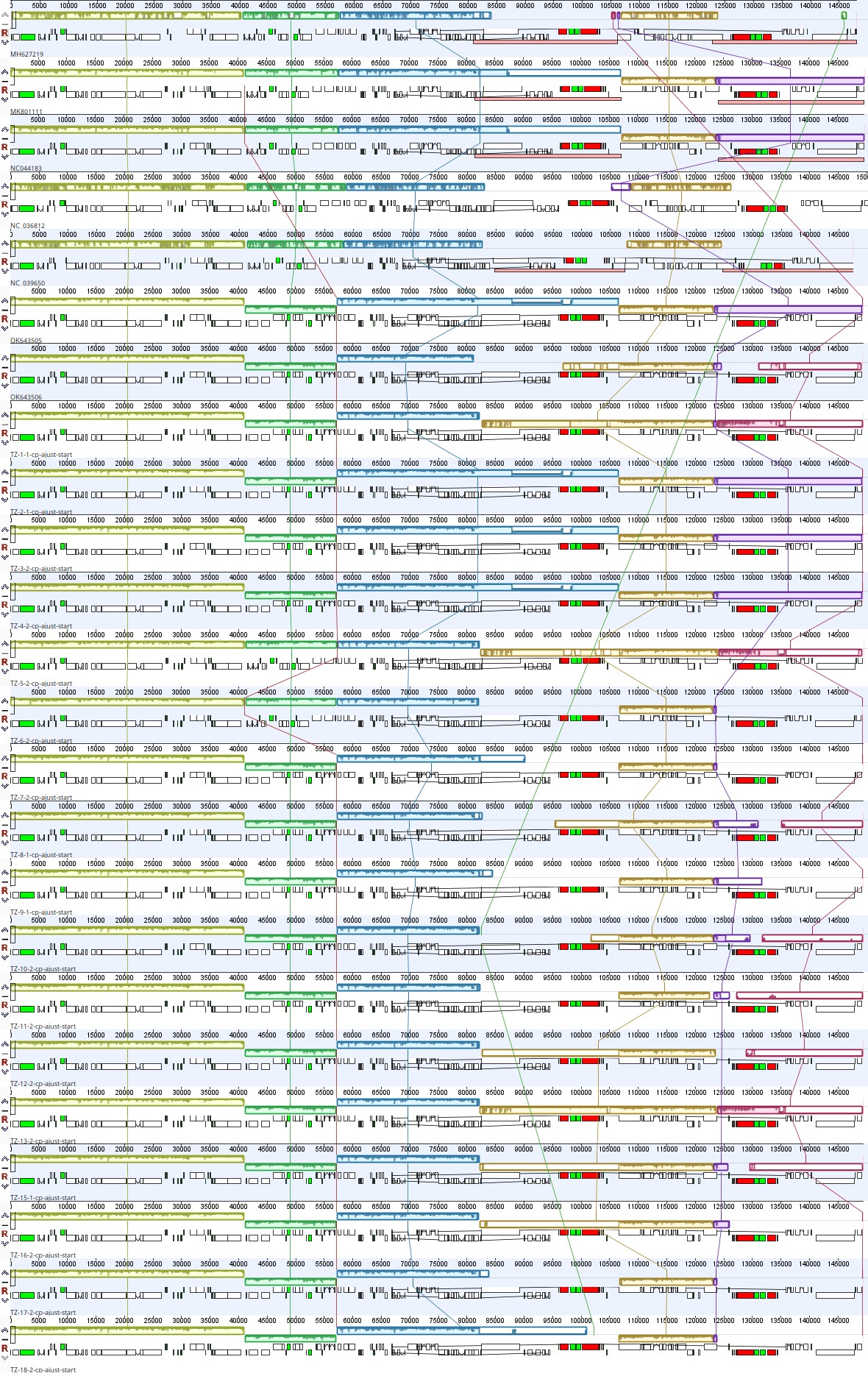

Supplement: Supplementary Figure 3 — A comparison of the whole plastid genomes of P. heterophylla using the Mauve algorithm. The red LCBs indicate syntenic regions, while the histograms within each block represent the degree of sequence similarity. rRNA, protein-coding, and tRNA gene annotations are denoted by red, white, and green boxes, respectively. [file Image_3.jpeg]

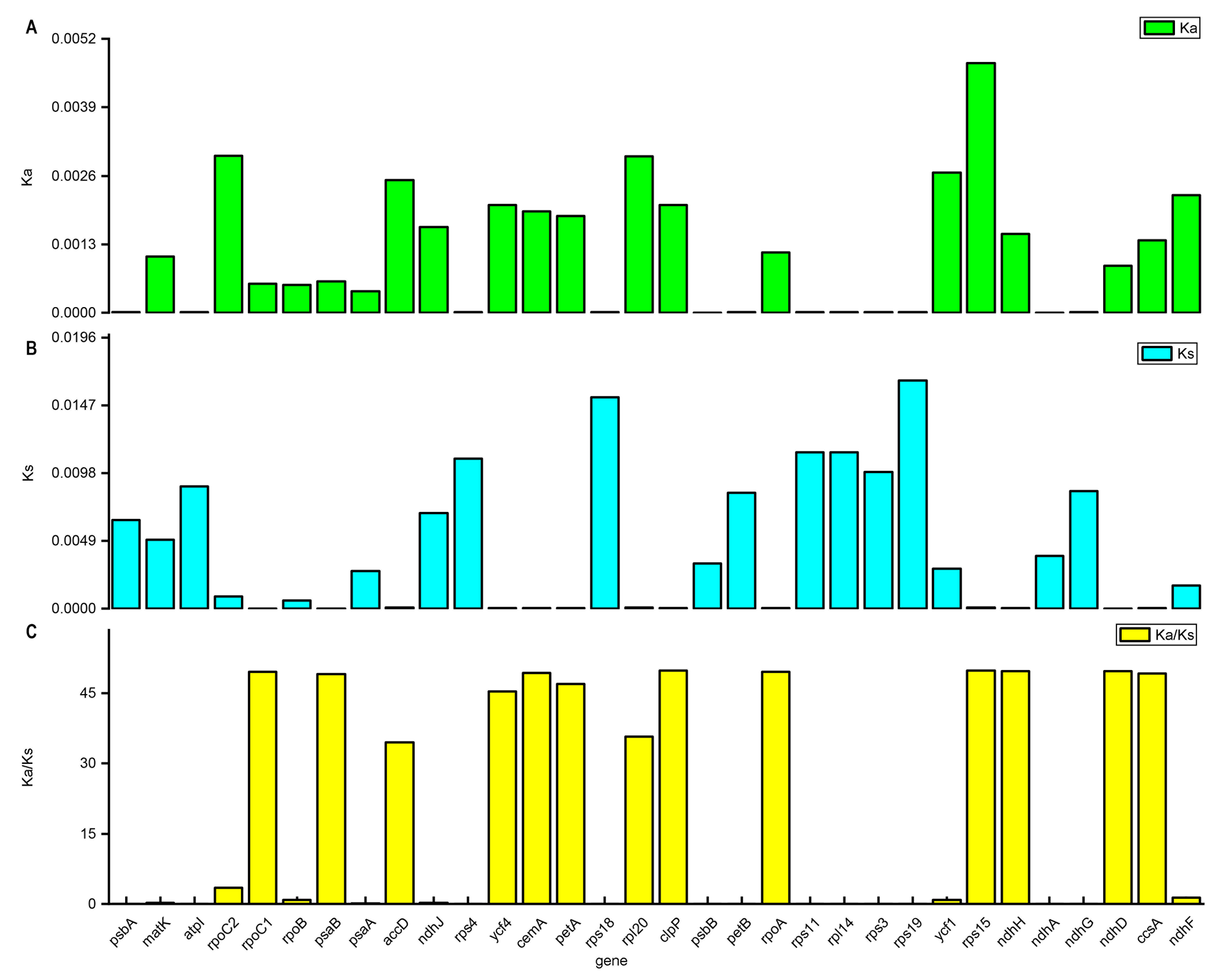

Supplement: Supplementary Figure 4 — Ka/Ks analysis of P. heterophylla chloroplast gnomes. (A) Ka, rate of nonsynonymous substitution; (B) Ks, rate of synonymous substitution; (C) Ka/Ks, rate of non-synonymous vs. synonymous substitutions. [file Image_4.tif]
